# Accelerated geroncogenesis in hereditary breast-ovarian cancer syndrome

**DOI:** 10.18632/oncotarget.7867

**Published:** 2016-03-02

**Authors:** Javier A. Menendez, Núria Folguera-Blasco, Elisabet Cuyàs, Salvador Fernández-Arroyo, Jorge Joven, Tomás Alarcón

**Affiliations:** ^1^ ProCURE (Program Against Cancer Therapeutic Resistance), Metabolism and Cancer Group, Catalan Institute of Oncology, Girona, Catalonia, Spain; ^2^ Molecular Oncology Group, Girona Biomedical Research Institute (IDIBGI), Salt, Catalonia, Spain; ^3^ Computational and Mathematical Biology Research Group, Centre de Recerca Matemàtica (CRM), Barcelona, Spain; ^4^ Unitat de Recerca Biomèdica, Hospital Universitari de Sant Joan, IISPV, Universitat Rovira i Virgili, Campus of International Excellence Southern Catalonia, Reus, Spain; ^5^ Institució Catalana d'Estudis i Recerca Avançats (ICREA), Barcelona, Spain; ^6^ Departament de Matemàtiques, Universitat Autònoma de Barcelona, Barcelona, Spain; ^7^ Barcelona Graduate School of Mathematics (BGSMath), Barcelona, Spain

**Keywords:** BRCA1, geroncogenesis, metabolism, cancer, metformin, Gerotarget

## Abstract

The geroncogenesis hypothesis postulates that the decline in metabolic cellular health that occurs naturally with aging drives a “field effect” predisposing normal tissues for cancer development. We propose that mutations in the cancer susceptibility genes *BRCA1/2* might trigger “accelerated geroncogenesis” in breast and ovarian epithelia. By speeding up the rate at which the metabolic threshold becomes “permissive” with survival and expansion of genomically unstable pre-tumoral epithelial cells, *BRCA* haploinsufficiency-driven metabolic reprogramming would operate as a *bona fide* oncogenic event enabling malignant transformation and tumor formation in *BRCA* carriers. The metabolic facet of *BRCA1* one-hit might involve tissue-specific alterations in acetyl-CoA, α-ketoglutarate, NAD^+^, FAD, or S-adenosylmethionine, critical factors for de/methylation or de/acetylation dynamics in the nuclear epigenome. This in turn might induce faulty epigenetic reprogramming at the “install phase” that directs cell-specific differentiation of breast/ovarian epithelial cells, which can ultimately determine the penetrance of *BRCA* defects during developmental windows of susceptibility. This model offers a framework to study whether metabolic drugs that prevent or revert metabolic reprogramming induced by *BRCA* haploinsufficiency might displace the “geroncogenic risk” of *BRCA* carriers to the age typical for those without the mutation. The identification of the key nodes that directly communicate changes in cellular metabolism to the chromatin in *BRCA* haploinsufficient cells may allow the epigenetic targeting of genomic instability using exclusively metabolic means. The validation of accelerated geroncogenesis as an inherited “one-hit” metabolic “field effect” might offer new strategies to therapeutically revisit the apparently irreversible genetic-hereditary fate of women with hereditary breast-ovarian cancer syndrome.

## Hereditary cancer and the “Angelina Jolie effect”: how high celebrity profile can have a major impact on worldwide biomedicine

The famous American film actress and director Angelina Jolie made public in 2013 [1] and 2015 [2] that, due to her high risk of developing cancer, she had undergone two surgical interventions: a double mastectomy (removal of both breasts), and later a bilateral salpingo-oophorectomy (removal of both ovaries and both Fallopian tubes). Three women in her family had died from the disease, including her mother, and DNA tests had revealed that Ms. Jolie carried a mutation in *BRCA1*, one of the cancer susceptibility genes linked to the so-called hereditary breast-ovarian cancer syndrome (HBOCS). Although hereditary tumors in women that, like Angelina Jolie, carry germline mutations in the *BRCA1* gene account for only a small percentage of breast and ovarian cancers (between 5% and 10%), the risk of developing the disease throughout their lifetime is much higher (up to 85%) than for women without the mutation.

The so-called “Angelina Jolie effect” has undoubtedly led to better social awareness of familial cancer syndromes and has resulted in an increase in the number of women inquiring about genetic screening to determine whether they are at a higher risk of developing breast and ovarian cancer because of mutations in the *BRCA1* and *BRCA2* genes [3-5]. Unfortunately, this “Jolie effect” has not translated, and will not in the short or mid-term, into significant changes in the few therapeutic options available to these women [6-11], namely: 1) undergo a very strict medical surveillance; 2) take chemopreventive therapy based on the selective estrogen-receptor modulator (SERM) tamoxifen -effective in a small percentage of cases, and with potential acute and long-term side effects- to reduce the risk of developing the disease; and 3) choose to have preventive surgery with removal of healthy breasts and ovaries, like Angelina Jolie, which could have a profound impact on the quality of life of the affected women. Thus, although other potential agents for chemoprevention, including the SERM raloxifene, the aromatase inhibitor exemestane, the poly (ADP-ribose) polymerase inhibitors veliparib and olaparib [12], and the RANK ligand inhibitor denosumab [13], might offer novel approaches for cancer prevention in *BRCA* mutation carriers, it is clear that there is an urgent need to offer alternative options to women inheriting *BRCA* mutations. These women are at high risk of developing breast and ovarian cancer that is generally very aggressive and difficult to treat, and which appears at a young age, sometimes making maternity an impossible goal to achieve. The question is: is it possible to revert, or at least modify, the apparently inevitable genetic-hereditary destiny of women carrying germline mutations in *BRCA1* and *BRCA2*?

## “Geroncogenesis”: Metabolic changes during aging as cancer enhancers

**Figure 1:**
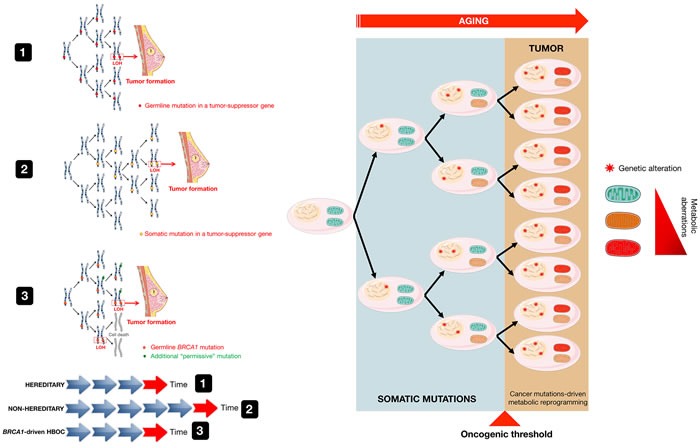
Hits and tumor formation in hereditary and nonhereditary carcinomas The mechanisms that underline genetic predisposition to cancer were originally clarified by Knudson, who hypothesized that germline mutations occur in one allele of a tumor suppressor gene followed by somatic inactivation, or loss of function, of the remaining normal allele through mutations, deletions, or epigenetic repression (defined as loss of heterozygosity [LOH]) [15] (model 1). The Knudson “two-hit” hypothesis has been largely validated in most forms of autosomal hereditable cancer and has been also extended to sporadic forms of cancer, albeit at a greater level of complexity [84]. Because in the nonhereditary scenario, the first somatic mutation might be expected to occur at a rate approximately equal to that of the second mutation in the hereditary cases, a “one-hit” clone is a precursor to the tumor formation in nonhereditary forms of cancer (model 2), whereas all cells are “one-hit” clones in hereditary cancer syndromes. *BRCA1*-driven HBOC syndrome, however, apparently contradicts the original “two-hit” theory conformed by other familial cancer syndromes, in which consecutive deletion of two alleles accelerates tumorigenesis. Cancer predisposition upon inactivation of a single *BRCA* allele relates to the so-called haploinsufficiency phenomenon associated with heterozygosity, which results in genomic instability in breast/ovarian epithelial cells. This in turn may promote additional genetic changes in *BRCA* heterozygous cells, including the acquisition of new mutations that will precede and be permissive with the loss of *BRCA* (e.g., *p53*, *ATM* and *CHK2*) (model 3). The requirement of this “extra-hit”, although incongruous from the viewpoint of familial tumorigenesis mediated by tumor suppressor genes such as *BRCA1* and *BRCA2*, appears to enable cancer-prone *BRCA* “one-hit” cells to evade the cell death processes that would otherwise occur upon loss of the remaining wild-type allele. Based on these models, cancer metabolic reprogramming is not the cause but rather the consequence of the mutations that originally generated malignancy and tumor growth (modified from original drawing published by Lindsay et al. [19]).

**Figure 2:**
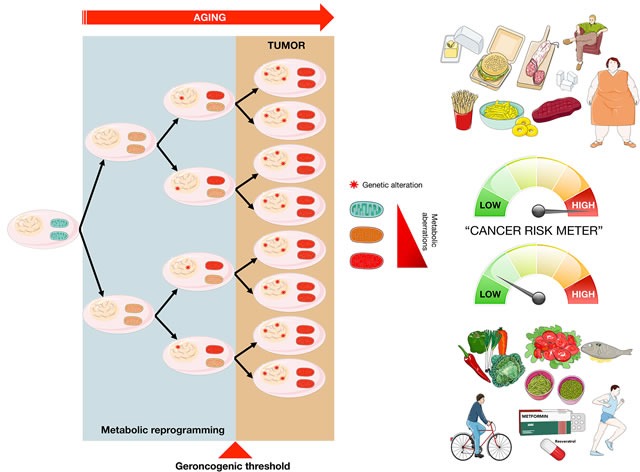
The “geroncogenic” hypothesis of carcinogenesis The natural deterioration in the function of cellular metabolism that occurs with age can be sufficient to generate an aberrant metabolic state in normal tissues capable of facilitating the independent or subsequent acquisition of cancer-driving genetic alterations (modified from original drawing published by Lindsay et al. [19]). While the model predicts that the geroncogenic risk could increase with excessive calorie intake and/or a sedentary lifestyle, it also suggests the possibility of countering this risk with low-calorie diets, physical exercise and by gerosuppressant agents such as metformin, rapamycin, and plant-derived polyphenols including resveratrol [85-87].

Among the known factors that contribute to cancer onset, the most important factor is age [14]. More than 60% of all cancers appear in people aged 65-70 years. Why? In 1971, Dr. Alfred Knudson proposed the “two-hit” hypothesis to explain the early onset at multiple sites in the body of an inherited form of cancer called hereditary retinoblastoma [15] (Figure 1, model 1). Knudson postulated that inheriting one *de novo* germline copy of a damaged gene present in every cell in the body, the so-called “first hit”, was not sufficient to enable retinoblastoma and other hereditary cancer syndromes to develop. The acquisition of a “second hit” to the remaining “healthy” copy in the gene pair was required and could occur somatically, which might rapidly lead to cancer because both copies of the normal tumor suppressor gene would be lost. Nonhereditary forms of the same cancer type would be poised to arise when two somatic mutations occurred in the same cell in susceptible tissue (Figure 1, model 2). Knudson´s hypothesis predicts that the chances for a germline mutation carrier to get a second somatic mutation at any of the multiple sites in their body are much greater than the chances for non-carriers to get two hits in the same cell. Thus, although the “first hit” germline mutation at the genotypic level is actually inherited in an autosomal dominant fashion, tumor suppressors apparently act recessively at the phenotypic level, *i.e.*, both alleles must be mutated/lost for cancer to develop. According to the widely accepted “two-hit” hypothesis of Knudson [15], tumors appear more frequently in the elderly because cells require time to accumulate enough oncogenic mutations to reach the minimum threshold of malignancy that is necessary for tumors to develop in a specific organ or tissue (Figure 1, *right panel*). Knudson's hypothesis of carcinogenesis, however, cannot explain why cancer risk is significantly reduced by calorie restriction or physical exercise, and also why an excess of calories and a sedentary lifestyle have the opposite effect [16]. Calorie restriction, up to 70% of *ab libitum* consumption, can completely halt tumor formation even in situations where chemical carcinogens would normally induce tumor formation in 100% of cases [17, 18; Figure 2].

Based on the classic Knudson model, the commonly observed metabolic changes in all types of cancer are not the cause but rather the consequence of the mutations that originally generated the aberrant tumor growth. Conversely, it has recently been postulated that tumor formation might not only depend on accumulating mutations in the genome, in a temporal manner, but also on the decline in the homeostasis or “metabolic health” that naturally occurs in our cells as we age [19-23]. Thus, the natural deterioration of the bioenergetic and biosynthetic machinery in normal cells could facilitate the genesis of an aberrant metabolic state favorable to support the processes of malignant transformation (Figure 2). This implies that, as we age, the possibility that genetic aberrations take place in a cell “pre-equipped” with a metabolism permissive for a process so bioenergetically and biosynthetically demanding as tumor transformation will increase. In this alternative model of carcinogenesis, known as geroncogenesis [19-21], the metabolic health of our cells would be a determinant factor to push the risk balance or “cancer risk-meter” to one side or the other. Thus, while the model predicts that the “geroncogenic” risk could increase with excessive calorie intake and a sedentary lifestyle, it also suggests the possibility of counteracting this risk with low-calorie diets and physical exercise. Realistically, the geroncogenic model adequately explains not only the observation that the major risk factor for cancer is age, but it is also consistent with the known associations between cancer prevalence and obesity and/or type 2 diabetes [24, 25], and the chemopreventive effect of molecules that maintain the metabolic health within cells, such as the polyphenol resveratrol [26, 27] or the anti-diabetic drug metformin [28-31].

## The enigmatic process of tumor formation in *BRCA* mutation carriers: still a topic of discussion 20 years after its discovery

It may seem surprising, but the mechanisms underlying tumor formation in women carrying heterozygous pathogenic mutations in one of the *BRCA* alleles inherited from one of the progenitors are still unknown despite more than 20 years passing since their discovery [32-35].

In hereditary familial cancer syndromes, individuals are termed heterozygous (having one or more dissimilar gene pairs) because they begin life with a germline mutation in one of the alleles linked to cancer susceptibility (*e.g.*, *BRCA1/2* alleles in the HBOCS) that is balanced by a normal counterpart. According to the “two-hit” postulate of Knudson [15], these individuals are predisposed to cancer since all of their cells have already sustained the first hit to cancer-linked genes (Figure 1, model 1). Thus, when the critically needed normal tumor suppressor gene that balances this germline mutation is lost at some point during the life of a carrier, a condition called loss of heterozygosity (LOH) occurs, which constitutes the most common molecular genetic alteration in human cancers. However, several factors make familial *BRCA* tumors not only extraordinarily complex but also enigmatically paradoxical. It is known that the wild-type alleles of the *BRCA* genes are lost in all the ovarian tumors and in a significant number of breast tumors that develop in women carrying a mutated allele [36]. Paradoxically, the complete absence of *BRCA* genes in adult human cells induces significant defects in cell proliferation that leads ultimately, in the majority of cases, to cell death. Moreover, this bi-allelic inactivation of *BRCA* observed in patient tumors results in early embryonic lethality when reproduced in animal models [37-39]. Globally, these studies show that: a) *BRCA* genes are essential for life since it is impossible to obtain live animals in the absence of this protein and, b) *BRCA* genes play an essential role in the maintenance of the genomic integrity of normal cells [40-42]. This raises the question, how can tumor cells survive with loss of both *BRCA1* alleles?

If we consider that *BRCA1/2* function as tumor suppressors in normal cells [36], *BRCA* tumor formation should presumably follow the “two-hit” theory, which would satisfactorily explain the accelerated carcinogenesis that occurs in other familial cancer syndromes [15]; that is, in those individuals with a genetic predisposition to cancer because of an inherited mutated allele in a tumor suppressor gene, it is considered that somatic LOH (i.e., the loss of the healthy allele) is a compulsory step for tumor formation and/or progression in those syndromes. However, to survive homozygotic inactivation of the *BRCA* genes during tumorigenesis, pre-tumoral cells in carriers must accumulate additional genetic alterations in other genome “guardian” genes, such as the tumor suppressor *p53*. Therefore, the requirement for an “extra-hit” to explain the process of familial tumorigenesis mediated by tumor suppressor genes such as *BRCA1/2* seems contradictory (Figure 1, model 3). The genomic instability that arises as a consequence of the inactivation of just one of the two *BRCA* alleles (a phenomenon known as haploinsufficiency) has been proposed as the mechanism that facilitates the acquisition of additional genetic alterations [40, 43, 44].

If the role of *BRCA* genes in a normal cell is to safeguard its malignant transformation, it would be expected that a 50% reduction in the expression of *BRCA* in women mutation carriers would coalesce with the emergence of a broad spectrum of tumors in all tissues. However, the loss of one half of the protective dosage of BRCA1/2 proteins increases the risk of tumor formation preferentially in hormone-dependent tissues such as mammary and ovarian epithelia. Although the enigma of this tissue-specificity for *BRCA* tumors [45] remains unresolved, numerous lines of evidence converge and suggest that the process of haploinsufficiency is capable of pre-initiating significant changes in the activation state of the hormonal signaling cascade (estrogen and progesterone receptors) that occurs naturally in those epithelial tissues, which in turn would lead to genomic instability phenomena and the acquisition of new mutations that will be “permissive” with the total loss of *BRCA1/2* (e.g., *p53*, *ATM* and *CHK2*) [46-48].

## Do women carrying *BRCA1/2* mutations undergo accelerated “geroncogenesis”?

*BRCA* heterozygosity seems able to alter, in a subtle but significant manner, the gene expression profiles, the activation status of intracellular signaling cascades (*i.e.*, hormone receptors), the differentiation degree, and the genomic stability of epithelial cells that, in every other way, are apparently completely normal, including their morphology within the mammary or ovarian tissue [49-53]. This suggests that breast and ovarian tumors could develop through a “silent” process in which accumulation of molecular changes from very early stages is caused by the chronic presence of just one “hit”, *BRCA1/2* haploinsufficiency, which would be sufficient to initiate tumor formation by “copying” the behavior of a cancer cell. In this scenario, it is important to note the following:

1. Epidemiological studies have demonstrated that physical exercise and absence of obesity in the teenage years are protective factors significantly associated with a later onset of breast cancer in Ashkenazi Jewish women, a population highly likely to inherit *BRCA* mutations [54-56]. Thus, while over 60% of women with a familial *BRCA1* mutation will develop breast cancer before the age of 70, “metabolically healthy” lifestyles might be able to significantly reduce the likelihood of pre-tumoral *BRCA1* haploinsufficient cells reaching the malignancy threshold.
2. Recent studies are beginning to reveal an until now unsuspected function of *BRCA1* in normal tissues, which is the regulation of cellular metabolism [57-62]. First, *BRCA1* regulates fatty acid biosynthesis both in adipose tissue and also in tumor cells [57-60]. Second, loss of *BRCA1* function induces a reprogramming of energetic metabolism in breast cancer cells [61]. Third, *BRCA1* regulates the process of cellular self-cannibalism known as autophagy [62], a complex molecular machinery that recycles damaged cellular constituents to mitigate the effects of metabolic stress.

**Figure 3:**
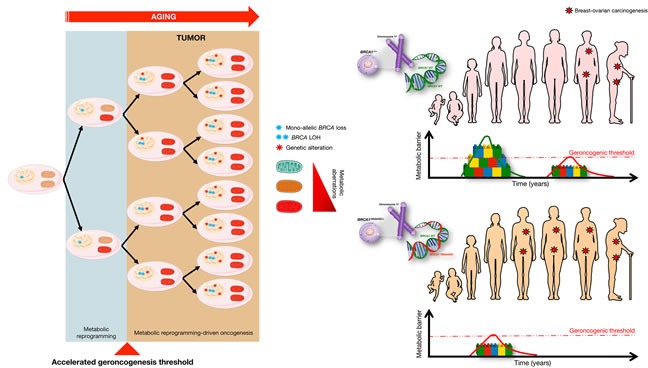
*BRCA1* haploinsufficiency induces “accelerated geroncogenesis” Women carrying mutations in the cancer susceptibility genes *BRCA1* and *BRCA2* might undergo a tissue- and cell type-specific process of “accelerated geroncogenesis” in breast and ovarian epithelia. *BRCA1* haploinsufficiency-driven metabolic rewiring of breast/ovarian epithelial cells to metabolic portraits capable of supporting the high bioenergetic and biosynthetic requirements of genomically unstable breast/ovarian epithelial cells to progress to a fully malignant phenotype might constitute an unanticipated and inherited form of metabolic reprogramming linked to increased risk of oncogenesis (modified from original drawing published by Lindsay et al. [19]). In this model of “accelerated geroncogenesis”, there would be a reduction in the time required for breast and ovarian epithelial cells to phenocopy a cancer-like metabolism, thus accelerating the rate at which the metabolic threshold becomes “permissive” with the survival and expansion of the pre-tumoral “one-hit” *BRCA*-deficient breast/ovarian epithelial cells. Although *BRCA* haploinsufficiency *per se* would significantly increase the “geroncogenic” risk, it would also make these patients more responsive to preventative and therapeutic strategies based on new drugs or approaches aimed to halt the aberrant metabolic reprogramming of breast/ovarian epithelial cells.

Although none of the above-mentioned studies were performed in models of *BRCA1* haploinsufficiency, it seems reasonable to propose that the tumor suppressor function of *BRCA1* might involve the participation of strictly metabolic mechanisms capable of restricting the emergence of a metabolic status compatible with malignant transformation. It is within this scenario that we postulate a slightly different version of the geroncogenic hypothesis in which women carrying mutations in *BRCA1* and *BRCA2* might undergo a process of “accelerated geroncogenesis” in mammary and ovarian epithelia (Figure 3). This new hypothetical framework assumes that:

- *BRCA1/2* haploinsufficiency is sufficient to produce significant and chronic cell-type-specific metabolic changes that would allow the earlier onset of an aberrant metabolic state compatible and “permissive” with the elevated genomic instability present in breast and ovarian epithelial cells in women carrying non-functional copies of *BRCA1/2*.
- The inheritance of a mutated allele in *BRCA1/2* might significantly reduce the time required for mammary and ovarian epithelial cells to become “pre-equipped” with an optimal metabolic performance to support a process so bioenergetically (Box 1) and biosynthetically demanding as tumor formation.
- By accelerating the rate at which the metabolic threshold is “permissive” with the survival and the expansion of the genomically unstable pre-tumoral cells, the metabolic facet of *BRCA* haploinsufficiency would operate as a true oncogenic event capable of provoking malignant transformation and tumor formation in women carrying the mutation.
- *BRCA1/2* haploinsufficiency would significantly increase the “geroncogenic risk” in these women, but also should make them responsive to new preventive and therapeutic strategies capable of restoring metabolic barriers (Box 1) incompatible with the genomic instability that occurs at an early stage in the epithelia of these women.
- The characterization of the “metabolic portrait” generated by *BRCA1/2* haploinsufficiency might inherently determine the preventive and therapeutic value of new anti-metabolic strategies in women carrying these mutations.

BOX 1 BRCA1 haploinsufficiency increases the metabolic potential of normal-like breast epithelial cells: A metabolic pre-adaptation suppressible by the mitochondrial poison metformin

To provide a preliminary proof-of-concept of the inherited metabolic facet of germline *BRCA* mutations in target tissues, we took advantage of isogenic pairs of nontumorigenic human breast epithelial cells (MCF10A) in which the knock-in of *185delAG* mutation in a single *BRCA1* allele leads to genomic instability [63]. Using a commercially available real-time assay on live cells (XFp Cell Energy Phenotype Test, Seahorse Bioscience) we measured both the mitochondrial and glycolytic activity in *BRCA1^+/+^* and *BRCA1^185delAG/+^* cells, and compared their baseline values with metabolic activity under stressed conditions to determine the so-called metabolic potential, i.e., the intrinsic cells' ability to respond to an energy demand. When simultaneously measuring the relative utilization of the major energy pathways under both basal and stressed conditions, we concluded that *BRCA1* haploinsufficiency was sufficient to notably augment the normal-like breast epithelial cells' ability to meet an energy demand *via* respiration and glycolysis. Importantly, this advantageous metabolic pre-equipment of *BRCA1* haploinsufficient cells was notably suppressed by pre-treating *BRCA1^185delAG/+^* cells with clinically relevant concentrations (1 μmol/L; 24 h) of the mitochondrial poison metformin. Indeed, metformin pre-treatment of *BRCA1^185delAG/+^* cells restored the baseline metabolic potential of *BRCA1^+/+^* parental cells, whereas the lower metabolic potential of *BRCA1^+/+^* parental cells remained unresponsive to the drug.

Because the metabolic potential can be viewed as the functional consequence of somatic or inherited germline mutations in terms of metabolic adaptations and reprogramming events that drive tumor malignancies, these findings support the notion that: a.) *BRCA1* haploinsufficiency might accelerate the geroncogenic trait that naturally occurs in the breast epithelium by enhancing the metabolic potential of epithelial cells; b.) new metabolic strategies (e.g., the mitochondrial poison metformin) aimed to halt the breast tissue- and epithelial cell-specific metabolic reprogramming imposed by *BRCA1* haploinsufficiency might be explored as preventive and therapeutic options in *BRCA1* carriers.

**Box:**
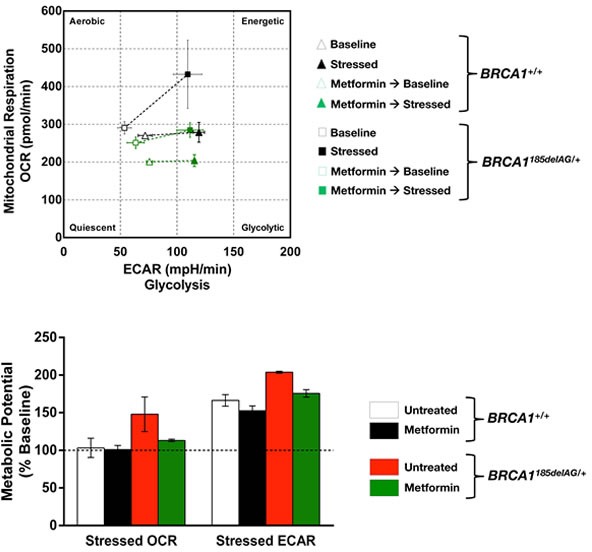
OCR: Oxygen Consumption Rates, in pMoles O^2^/min ECAR: Extracellular Acidification Rate, in mpH/min. ECAR data were not artificially altered by changes in pH values, as in fact closely paralleled changes in PPR (normalized Proton Production Rate, in nmol H+/min) values (data not shown)

## Accelerated geroncogenesis in *BRCA1* carriers: A proof-of-concept framework to validate aberrant metabolism as a *bona fide* “cancer trait”

The clinical relevance of alterations at the “one-hit” level to carcinogenesis is highlighted by the fact that a very high number of patients with a heritable cancer syndrome, including those suffering HBOCS, will develop cancer [64]. This supports the notion that specific molecular features in otherwise phenotypically normal cells in target tissues might be directly associated with actual cancer development. Indeed, the fact that germline variants in *BRCA1/2* are inherited in an autosomal dominant manner strongly indicate that molecular features gathered at the cellular level in those individuals are directly associated with cancer initiation, which may provide further insights about the development of specific cancers (e.g., breast and ovarian carcinomas). Moreover, alterations in normal-like cells from target tissues in patients representing different forms of heritable cancer such as HBOCS might be able to capture not only changes that are unique to a specific pathway(s) for HBOCS, but they might also involve “global changes” that can be shared, whether systemic or at the target site, not only by different forms of heritable cancers at the tumor level but also by sporadic forms of cancer. Because these global changes can be defined as the cancer trait, the metabolic characterization of otherwise normal breast epithelial cells from one-hit *BRCA* carriers (which do not include most of the confounding secondary effects that take place at the two-hit tumor stage) should illuminate the earliest metabolic changes (and, consequently, therapeutically valuable metabolic targets) that represent “engine drivers” in sporadic forms of cancer also, which might enable a better prioritization and validation of relevant metabolic biomarkers and optimal metabolic targets at the “two-hit” tumor stage.

We propose that metabolomic analyses of phenotypically normal, one-hit *BRCA* breast/ovarian epithelial cells might capture early metabolic changes that could serve as risk biomarkers and targets for preventive agents relevant to the management not only of HBOCS, but also of a much larger group of cases of sporadic breast and ovarian cancers. By providing the precise metabolic mechanisms for tumor initiation in a tissue-dependent manner, breast/ovarian epithelial cells from *BRCA1* carriers can therefore be viewed as fertile models not only for understanding the basic metabolic mechanisms of oncogenesis, but also as idoneous clinical scenarios for how to realize the promise of personalized therapeutic approaches to metabolic cancer prevention and therapy in a much larger group of sporadic cancers.

Nevertheless, we should acknowledge that there is no preventive option capable of successfully reverting the genetic-hereditary fate in women that carry mutations in cancer-predisposing *BRCA1/2* genes beyond strict surveillance or preventive mastectomy, the extreme alternative chosen by Angelina Jolie. Assuming that inherited mutations will irreversibly lead to cancer in these women, our model of “accelerated geroncogenesis” offers an alternative scenario, testable from a therapeutic point-of-view, where energetic behaviors or new drugs that prevent or revert the pro-tumoral metabolic alterations induced at an early stage by haploinsufficiency of *BRCA1/2*, could restore metabolic barriers for the processes of genomic instability that occur in the epithelia of these women. This would displace their “geroncogenic risk” to the age typical for women without the mutation. Thus, our model *a priori* predicts that “metabolically healthy” lifestyles (e.g., daily physical exercise, intermittent fasting) and, more specifically, the use of anti-metabolic drugs to stop or revert the metabolic reprogramming induced by *BRCA1/2* haploinsufficiency in the epithelia of target tissues, could be new effective strategies for the early prevention and therapeutic treatment by displacing the “geroncogenic risk” of *BRCA* carriers. In this regard, it should be noted a significant increase in the risk of developing diabetes after a breast cancer diagnosis appears to occur in women with *BRCA1/2* mutation [65]. Because a high Body Mass Index (BMI), which is an indicator of high body fatness, and the use of chemotherapy, compounded this increased risk in *BRCA1/2* carriers, further investigation should clarify if, in addition to cell-autonomous mechanisms, the metabolic facet of *BRCA1* one-hit also involves significantly imbalanced non-cell-autonomous environmental factors (e.g., IGF-I, estrogens) compared to women treated for breast cancer who do not have a *BRCA* mutation [66-68].

## Accelerated geroncogenesis in *BRCA1*-driven HBOCS: Metabolism as the molecular bridge connecting irreversible germline genetic alterations with reversible epigenetic reprogramming

**Figure 4:**
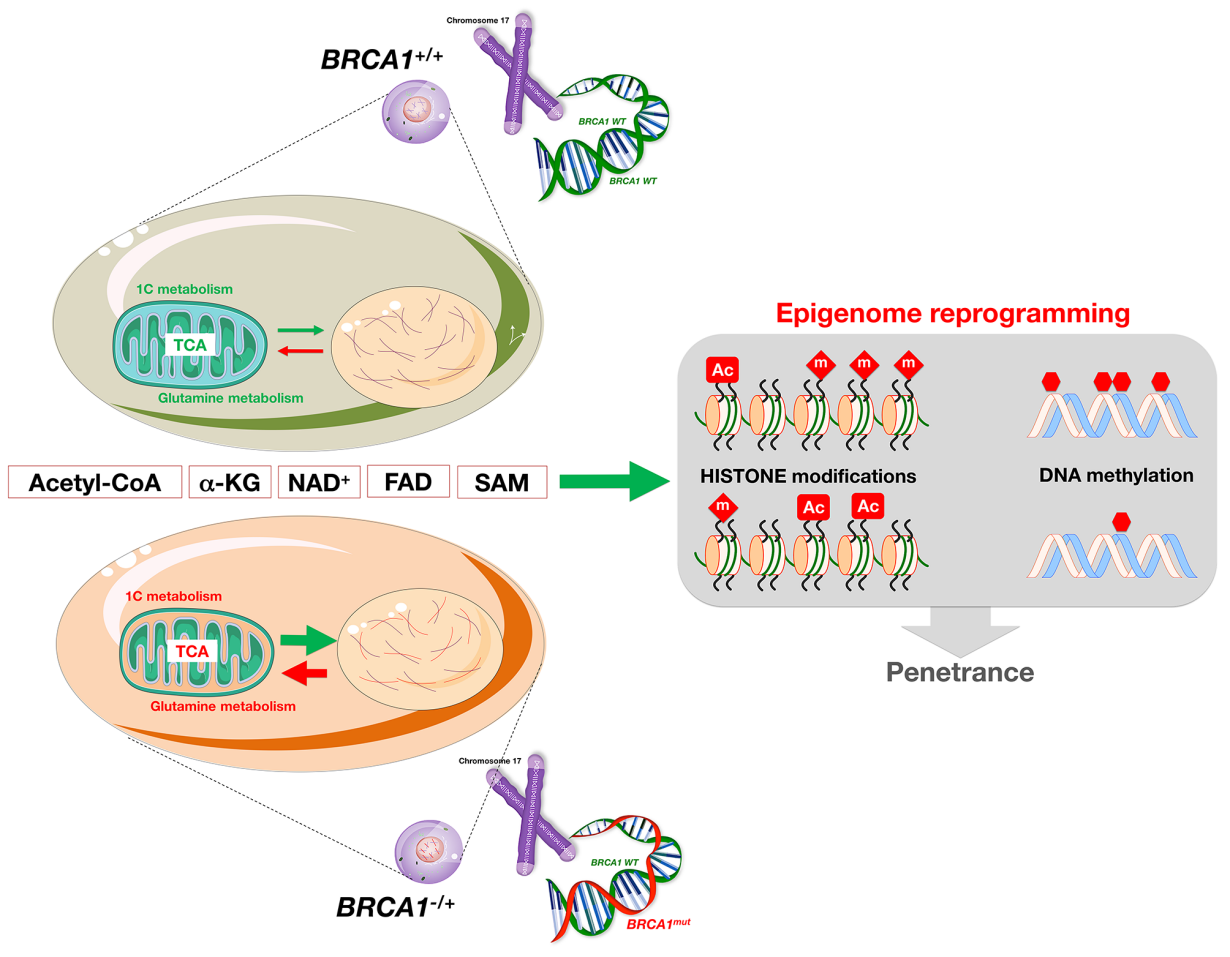
Metabolic regulation of epigenetics: The reprogramming dimension of *BRCA1*-driven accelerated geroncogenesis Haploinsufficiency for *BRCA1/2* can lead to cell-type-specific mitochondrial functioning that invokes a significantly altered mitochondria-to-nucleus retrograde response in breast/ovarian epithelia. This response may induce relevant changes to the nuclear epigenome *via* alteration of key metabolic co-factors closely associated with the processes of active de/methylation or de/acetylation (e.g., acetyl-CoA, α-ketoglutarate [α-KG], NAD^+^, FAD, and S-adenosylmethionine [SAM]), which might increase the penetrance of tumor susceptibility, but may also illuminate new interventions that can reverse the epigenetic effects of metabolic reprogramming to decrease cancer risk associated with germline alterations in BRCA1/2 tumor suppressor genes.

Accumulated evidence indicates that some individuals with *BRCA1/2* germline variants can survive to an elderly age without developing cancer, while others never develop cancer. Among those who develop cancer, the age of onset and the presentation of the disease, also varies. Although no clear explanation exists for these observations, it has been generally thought that exposure to certain (micro)environmental factors ultimately determines the penetrance of tumor suppressor gene defects by inducing “second hits” in the normal allele or promoting the acquisition of additional genetic alterations that are required for multistage carcinogenesis. The developmental origins of health and disease (DOHaD) hypothesis provides an alternative model whereby environmental exposure during development increases susceptibility to cancer in adulthood not by inducing genetic mutations but by reprogramming the epigenome [69]. In the DOHaD framework, *BRCA1* haploinsufficiency-driven altered metabolism during development might cooperate to increase the penetrance of the defective *BRCA1* gene by significantly altering the epigenetic machinery in the target cells during breast organogenesis, which, in contrast to most organs that are fully developed at birth, takes place postnatally in adolescence and in adulthood. One possibility is that *BRCA1* carriers might develop early aberrant patterns of histone and DNA methylation *via* the ability of *BRCA1* to regulate the abundance of co-factors synthesized in mitochondria, which regulate genomic DNA methylation and chromatin structure/dynamics within the nuclear genome (e.g., acetyl-CoA, α-ketoglutarate, NAD^+^, FAD, or S-adenosylmethionine) (Figure 4). If a breast/ovarian epithelial tissue-specific alteration in these metabolic co-factors, which are associated with processes of active de/methylation or de/acetylation, occurs because of *BRCA1* haploinsufficiency, faulty epigenetic reprogramming may be imparted at the “install phase” that directs cell-specific differentiation during the inextricably unidirectional program of breast/ovarian development. A metabolically-driven pre-installation of defective epigenetic programs on the genome of these cells might aberrantly pre-adapt the developing tissues to the future adult environment, which can adversely affect cancer risk during developmental windows of susceptibility, thus ultimately determining the penetrance of *BRCA* defects.

Although epigenetic changes might be detected in at-risk tissues prior to the development of tumors in *BRCA1* carriers [67, 68], thus confirming that the effects of *BRCA* deficiency-driven epigenetic reprogramming are apparent in the “normal tissue” before the development of disease, it remains to be elucidated whether the effects of one-hit *BRCA1/2* germline mutations on the epigenetic landscape of cells at target tissues are a direct consequence of altered mitochondrial metabolic status and/or changes in one-carbon metabolism [73-74] in *BRCA1* haploinsufficient breast/ovarian epithelial cells. Crucially, however, the metabolic nature of this ability to reprogram the epigenome intrinsically holds promise for new interventions that can target the metabolo-epigenetic phenotype [75] of accelerated geroncogenesis. In contrast to germline alterations in *BRCA1/2* genes, which are generally irreversible, the identification of the key nodes that directly communicate changes in cellular metabolism to the chromatin state in *BRCA* haploinsufficient breast/ovarian epithelial cells may revolutionarily allow the epigenetic targeting of genomic instability by using exclusively metabolic means. Moreover, if the metabolic facet of *BRCA1* one-hit involves tissue-specific alteration of the signaling emanating from mammalian target of rapamycin (mTOR), the recently uncovered *BRCA1* haploinsufficiency-induced senescence (HIS) [76] might be understood as an accelerated form of mTOR-driven “geronconversion” [77-83] specific to epithelial cells. Experimental confirmation of the accelerated nature of cell- and tissue-specific geroncogenesis in the breast and ovarian epithelia of *BRCA* carriers should provide us with unanticipated metabolic tools to therapeutically revisit the apparently irreversible genetic-hereditary fate of women at high risk of suffering breast and ovarian cancer.
